# Dr. Leon F. Harvey

**Published:** 1913-01

**Authors:** 


Dr. Leon F. Harvey, Jefferson, 1859, died recently at New Rochelle. He was born in Buffalo in 1837, and practiced in Baltimore and Buffalo, chiefly, however, as a dentist. About 1887, he retired from dental practice and, as a matter of philanthropy and a token of his interest in medicine, devoted much time for several years to the management of the Fitch Provident Dispensary in Buffalo. Many will remember with gratitude not only his services to charity but his uniform courtesy and kindness to students and attending physicians.

